# Green synthesis, characterization, and hepatoprotective effect of zinc oxide nanoparticles from *Moringa oleifera* leaves in CCl_4_-treated albino rats

**DOI:** 10.1016/j.heliyon.2024.e30627

**Published:** 2024-05-03

**Authors:** Hossam S. El-Beltagi, Marwa Rageb, Mahmoud M. El-Saber, Ragab A. El-Masry, Khaled M.A. Ramadan, Mahmoud Kandeel, Ahlam Saleh Alhajri, Ali Osman

**Affiliations:** a Agricultural Biotechnology Department, College of Agriculture and Food Sciences, King Faisal University, Al-Ahsa, 31982, Saudi Arabia; b Biochemistry Department, Cairo University, Giza, 12613, Egypt; c Biochemistry Department, Zagazig University, Zagazig, 44511, Egypt; d Biochemistry Unit, Genetic Resources Department, Desert Research Center, Cairo, 11753, Egypt; e Central Laboratories, Department of Chemistry, King Faisal University, Al-Ahsa, 31982, Saudi Arabia; f Department of Agricultural Biochemistry, Ain Shams University, P.O. Box 68, Hadayek Shobra, Cairo, 11241, Egypt; g Department of Biomedical Sciences, College of Veterinary Medicine, King Faisal University, Al-Ahsa, 31982, Saudi Arabia; h Department of Pharmacology, Kafrelsheikh University, Kafrelsheikh, 33516, Egypt; i Food Science and Nutrition Department, College of Agricultural and Food Science, King Faisal University, Al-Ahsa, 31982, Saudi Arabia

**Keywords:** Green synthesis, ZnO-Nanoparticles, Hepatoprotective effect, Liver damage, CCl_4_, TEM, DLS

## Abstract

Hepatotoxin carbon tetrachloride (CCl_4_) causes liver injury. This research aims to create ZnO-NPs using green synthesis from *Moringa oleifera* (MO) leaves aqueous extract, and chemically prepared and confirming the synthesis by specialized equipment analysis. The sizes formed of ZnO-NPs were 80 and 55 nm for chemical and green methods, respectively. In addition, to study their ability to protect Wistar Albino male rats against oxidative stress exposed to carbon tetrachloride. MO leaf aqueous extract, green synthesized ZnO-NPs, and ZnO-NPs prepared chemically at 100 and 200 mg/kg BW per day were investigated for their hepatoprotective effects on liver enzyme biomarkers, renal biomarkers, antioxidant enzymes, lipid peroxidation, hematological parameters, and histopathological changes. Compared to the control group, all liver and kidney indicators were considerably elevated after the CCl_4_ injection. However, the activity of antioxidant enzymes in the liver was significantly reduced after the CCl_4_ injection. These outcomes indicate that MO leaf aqueous extract, greenly synthesized ZnO-NPs, and ZnO-NPs chemically prepared can restore normal liver and kidney function and activity, as well as hematological and antioxidant enzymes. The highest impact on enhancing the hepatoprotective effect was recorded for rats that received green synthesized ZnO-NPs. The increased drug delivery mechanism of green synthesized ZnO-NPs resulted in a higher protective effect than that of MO leaf aqueous extract.

## Graphical abstract

**Figure:**
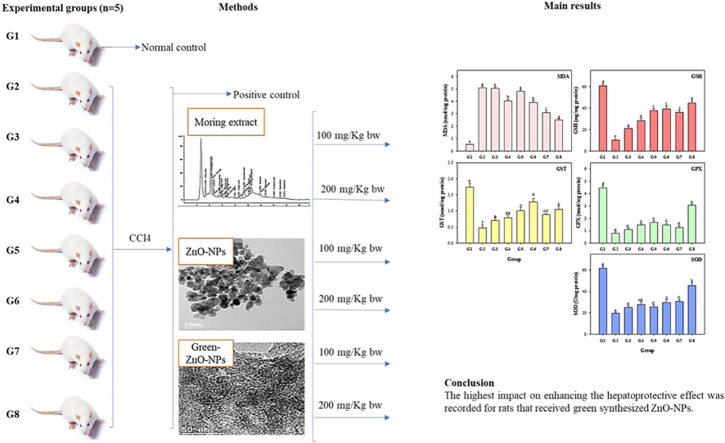

## 1 Introduction

Globally, hepatic damage caused by various environmental pollutants and liver-toxic substances is recognized as a critical medical condition. Hepatic damage resulting from alcohol and drug misuse affects many people worldwide [1]. Due to its vital function in the breakdown of pharmaceuticals and toxins, the liver is the primary organ that is first affected by chemical substances [2]. Numerous medicinal plants and their formulations are used for liver disorders in both ethnomedical practices and traditional medicine [3]. *Moringa oleifera* (MO) is a perennial arboreal species that grows throughout many regions of Asia, Africa, and North America [4]. This plant is well-known for its nutritional and medicinal properties [5]. Secondary metabolites can be located in the leaves, including vitamins, lignins, stilbenes, battalions, terpenoids, phenolic acids, alkaloids, and amines [6]. MO has been shown to be effective in mitigating inflammation, combating infections, enhancing antioxidant activity, suppressing cancerous growth, and promoting hepatic health [7]. MO is extensively used to treat liver diseases. The pharmacological effects of MO leaves may be attributed to their high content of bioactive substances [8]. The hepatoprotective effects of ethanolic [[9], [10], [11], [12]] and methanolic [13,14] extracts of MO leaves against liver damage induced by CCl_4_ were evaluated. Recently, nanotechnology-based therapies have emerged as novel and promising alternatives to conventional treatment. The field of nanotechnology is expanding rapidly, focusing on creating, manipulating, and using materials with dimensions between 10 and 500 nm for several medicinal treatments and drug-delivering systems [15]. The application of nanoparticles (NPs) in modern technology has expanded dramatically, yet the risk to human health cannot be assessed due to a lack of evidence. NPs began to gain interest because of their chemistry, small size, non-biodegradability, and reactive surfaces. They can easily disperse in the environment without any consequences [16]. Nanotechnology use has increased globally because to its application in diagnosis, drug delivery systems, the food sector, paints, cosmetics, electronics, and sports [17]. *In vitro* and *in vivo* studies demonstrated that many plant extracts and metallic nanoparticles have significant biological activities. Therefore, using plant extracts in the metallic nanoparticles green synthesis is expected to result in a synergistic effect, potentially leading to more convincing biological activities [18,19]. Therefore, combining plant extracts with metallic nanoparticles can synthesize a new nanomaterial with enhanced and exciting biological applications. CCl_4_ is a well-known hepatotoxic substance that induces swift liver damage, starting with the accumulation of fat in the liver (steatosis) and progressing to the death of liver cells in the central region of the liver lobule (Centrilobular necrosis). Chronic liver injury is induced by the prolonged infusion of CCl_4_, making it a widely used model for the development of hepatic fibrosis [20]. In recent years, green methods of production have been employed to create nanoparticles (NPs), such as zinc oxide (ZnO), Fe_3_O_4_, CuO, and Se. Many biological processes make use of these NPs [21]. ZnO-NPs were easily manufactured, environmentally benign, and nontoxic among the numerous metal oxide nanoparticles that were examined [22]. Previous studies have shown that ZnO-NPs can reduce oxidative stress, apoptosis, and inflammation [23,24]. It has been proven that ZnO-NPs inhibits lipid peroxidation and protects the liver from free radical damage [24]. It can be used to deliver an extensive range of medications into multiple organs, including the liver, brain, spleen, lungs, and lymphatic system [25,26]. Previous research has shown strong evidence for the role of ZnO-NPs in preventing the development of hepatic steatosis by activating AMPK signaling [27]. Therefore, numerous studies were conducted to investigate the biosynthesis of ZnO-NPs [[28], [29], [30]]. Furthermore, the MO plant has been extensively studied for synthesizing ZnO-NPs as stabilizing and reducing agents [[31], [32], [33], [34]]. Researchers are actively concentrating on green chemistry in the synthesis of ZnO-NPs, particularly in their hepatoprotective functions against liver injury [35]. Given this, the present investigation investigates the hepatoprotective effects of ZnO-NPs synthesized by MO aquatic extract on CCl_4_-treated albino rats.

## 2 Materials and methods

### 2.1 Botanicals and chemical compounds

Moringa leaves were collected in April 2023 from the Desert Research Center, Cairo, Egypt. It was identified by the Egyptian Moringa Society, National Research Center, Cairo, Egypt as *Moringa oleifera*. The compounds utilized in this investigation were acquired from Merck (KGaA, Darmstadt, Germany).

### 2.2 Phytochemical screening

Several gravimetric methods were used to establish how much of the necessary components, such as flavonoids [36], steroids [37], terpenoids [38], alkaloids [39], tannins [40], and saponins [41], were present in the leaves of MO.

### 2.3 Aqueous extract preparation

MO leaf aqueous extract (10 % w/v) was made by soaking 10 g of crushed leaves in one hundred mL of DW for 24 h with stirring by an orbital shaker (Thermo Fisher Scientific, Inc). The extract underwent filtration using Whatman No. 4 filter paper, and centrifuged for 15 min at 5000 rpm; the crude extract was obtained using lyophilization and afterward preserved at a temperature of 4 °C until it was ready for use [30,42].

### 2.4 Moringa oleifera leaf aqueous extract characterization

#### 2.4.1 Total phenolic substances (TPCs) estimation

TPC content was measured using the Folin–Ciocalteu assay [43]. Then, 0.3 mL from the sample (1000 μg/mL) was combined with 1.2 mL of a 10 % v/v Folin–Ciocalteau buffer and 1.5 mL from Na_2_CO_3_ (7.5 % w/v). After incubating the reaction mixture for 1 h, the absorbance at 765 nm was determined. A calibration curve was produced using a standard solution of gallic acid in concentrations varying from 20 to 200 μg/mL. Results per gram of extract were obtained as mg of gallic acid equivalents (GAE) from the following equations: y = 0.001x + 0.0563 (R2 = 0.9792), where y is the absorbance and x is the amount of gallic acid in μg/mL.

#### 2.4.2 Total flavonoid content (TFC) estimation

TFC was determined by employing the AlCl_3_ colorimetric technique [44]. In summary, a blend was created by combining 1.5 mL of methanol, 0.1 mL of potassium acetate in a 1 M solution, 2.8 mL DW, and 0.1 mL of a 10 % AlCl_3_ solution with 0.5 mL of the tested sample (1000 μg/mL). The solution was sustained at 25 °C for 30 min. The absorbance of solutions at 415 nm was estimated utilizing a spectrophotometer. Quercetin (20–200 μg/mL) was used to create a standard curve. The calibration curve was employed to assess the TFC using quercetin equivalent (QE). The quercetin calibration equation is y = 0.0012x + 0.008 (R2 = 0.944), where x is the quercetin concentration in g/mL and y is the absorbance.

#### 2.4.3 Antioxidant activity

Extract AA was tested using the DPPH free radical scavenging test [45]. In summary, a 2.9 mL aliquot of 0.1 mM DPPH (dissolved in methyl alcohol) was combined with varying concentrations of the extract, namely, 500, 1000, 1500, or 2000 μg/mL. The reaction was permitted to proceed for 30 min at 25 °C under conditions of minimal light exposure. Absorbance was recorded at 517 nm. The following equation was used to compute the radical scavenging capacity of DPPH:

Inhibition(%)=[controlabsorbance−sampleabsorbance)/controlabsorbance]x100.

#### 2.4.4 Phenolic compound identification by HPLC

The chemical constitution of phenolic compounds in an aqueous extract of MO was analyzed using the HPLC analytical method, as previously mentioned in an earlier report [46]. Detecting phenolic compounds was conducted using the Agilent 1260 Infinity HPLC system (5301 Stevens Creek Blvd., Santa Clara, CA 95051, United States). The utilized HPLC has a G1311C-QuatPump, a G1329B-autosampler, a G1316A column heater, a G1314F variable wavelength detector, and a G1322A degasser. The Agilent HPLC ChemStation 10.1 edition was used on a Windows 7 operating system for instrument management and data analysis. The column employed in this study was an Agilent Zorbax C18 column with dimensions of 5 m, 4.6 mm in diameter, and 150 mm in length. The injection volume was 50 μL, and the mobile phase composition was composed of two components: A, a mixture of 70 % methanol and 30 % water; B, 100 % methanol. The phenolic compounds were identified by comparing their retention times to those of an external standard.

#### 2.4.5 Gas chromatography-mass spectrometry

A solution of hexane 99 %, with a stabilizer content of 0.5%–1.0 % ethanol, was employed to dissolve approximately 1 g of MO lyophilized crude extract. Then, the extract was evaporated for GC-MS testing. The obtained hexane extract from the MO sample was subjected to analysis using the Agilent Technologies-7820A GC instrument combined with mass spectrometry (GC-MS). The experimental setup consisted of an Agilent Technologies GC-MS capillary column, namely, the HP-5MS variant, with dimensions of 30 m in length, 0.25 mm in inner diameter, and 0.25 m in film thickness. This column consisted of a 5 % diphenyl and 95 % dimethyl polysiloxane mixture. It was coupled to Agilent Technologies GC-MS mass spectrometer, specifically the model 5977MSD. A device using electron ionization with an ionizing energy of 70 electron volts (eV) was utilized. The carrier gas in this study was helium gas, with a purity of 99.99 %. The split ratio utilized was 50:1, with an injection volume of 1 mL. The injector temperature was set at 60 °C, while the ion source temperature was maintained at 250 °C. Moreover, the transfer line was kept at 240 °C, while the ion supply was also set to a temperature of 240 °C. The ionization mode employed was electron impact at an energy level of 70 eV. The scan period for the analysis was set at 0.2 s, with a scan interval of 0.1 s. The fragment size range was 40–600 Da. The components in the extracts were initially identified with peaks in mass spectra using Computer Wiley MS libraries and validated by comparing the two datasets [47].

### 2.5 Chemically synthesis of ZnO-NPs

In this experiment, zinc nitrate, sodium hydroxide, and sodium borohydride are employed as precursor materials. The synthesis procedure of ZnO-NPs involved using sodium borohydride as a stabilizing agent, following the methods described in Ref. [48] with some modifications. Calculations should be conducted after the reaction and the creation of zinc hydroxide to get the desired size. In summary, 100 mL of distilled, deionized water was measured and combined with 2.79 g of Zn (NO_3_)_2_.6H_2_O. The resulting mixture was vigorously agitated for 1 h. Then, the NaOH (10 %) was incrementally added in droplets. After dispersing the zinc nitrate, 1 mL of NaBH_4_ (1 %) was added and agitated for 12 h to obtain a low nanosized solution. After letting the precipitate settle, it was filtered using suction and repeatedly rinsed with distilled, deionized water. Subsequently, the precipitate was dried at 80 °C in the oven. ZnO was created after Zn (OH)_2_ had first been generated. Muffle calcination was conducted at 500 °C for 3 h in order to achieve a reduced size.

### 2.6 Green synthesis of ZnO-NPs

**Fig. 1:**
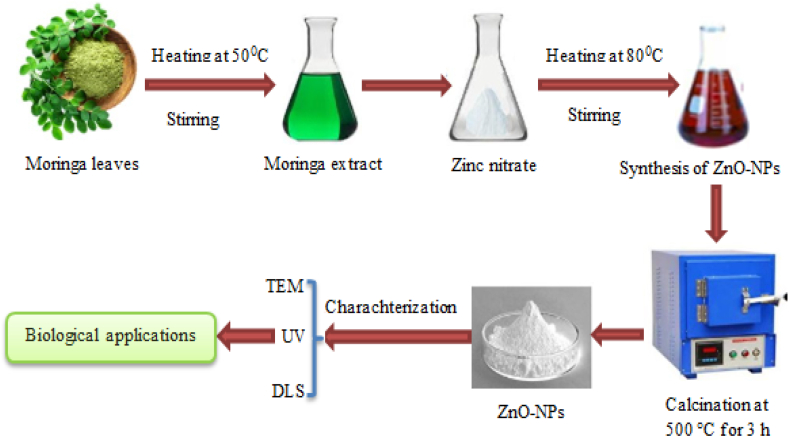
Green synthesis process of ZnO-NPs using moringa aqueous leaf extract.

In total, 10 g of MO specimens was produced in the greenhouse located at the DRC in Cairo, Egypt. The gathered specimens were subjected to a cleaning process using distilled water, and the surfaces of the leaves were carefully sterilized by using rubbing alcohol. The steps of the green synthesis process of ZnO-NPs using moringa aqueous leaf extract are provided in Fig. 1. The leaves were subjected to a 40-min treatment at 50 °C using 100 mL of distilled water. Whatman 41 filter paper (Merck KGaA, Darmstadt, Germany) was used to filter the extract. The filtrate was kept in a dry, cool environment. Then, 10 mL of MO leaf extract and 90 mL of 0.05 mM Zn(NO_3_)_2_.6H_2_O solution were used to create ZnO-NPs. The mixture was continuously stirred while being heated at 80 °C for 30 min. Nanoparticle production was evidenced by the existence of white particles. Following the centrifugation process, the residual pellets derived from the extract were gathered, subjected to desiccation, and subsequently exposed to calcination at 500 °C for 3 h [30,49,50].

### 2.7 Analysis of naturally occurring and synthetic ZnO-NPs

#### 2.7.1 Transmission electron microscopy (TEM)

The size and morphology of ZnO-NPs can be evaluated experimentally using TEM, specifically employing JEOL's JEM-2100 instrument. A small amount of colloidal solution was carefully placed onto a copper grid coated with carbon and a 400 mesh to prepare the specimens for TEM investigation. Subsequently, the solvent was left to evaporate naturally at ambient room temperature. Image J software was used to determine the mean particle size of NPs.

#### 2.7.2 Ultraviolet–visible (UV-VIS) spectra

The synthesis of zinc oxide nanoparticles in an MO leaf aqueous extract was tracked using the UV-VIS capability of a Shimadzu spectrophotometer. UV–Vis spectra were collected between 300 and 700 nm in wavelength.

#### 2.7.3 Dynamic light scattering (DLS) zeta potential and particle size analysis

Malvern zetasizer nano instrument was employed to assess both the size of particles and their zeta potential. Samples in solution form were located in cuvettes to mitigate sample transparency until the reading error was reduced before being placed inside the device. The apparatus called for particles of a specific diameter, and homogeneity was measured using the polydispersity index (PDI), which ranges from 0 to 1, with greater homogeneity observed at lower values.

### 2.8 Hepatoprotective impact

This work aims to explore the hepatoprotective impact of aqueous extract of MO leaves, as well as green and chemically synthesized ZnO-NPs, toward liver damage caused by CCl_4_ in male Wistar rats. The present study was conducted at an animal facility located within the Forensic Medicine Department, Faculty of Medicine, Zagazig University, Zagazig, Egypt.

#### 2.8.1 Animal model

In this investigation, a total of 40 mature male albino rats of the Wistar strain, ranging between 150 and 170 g, were generously contributed by the Organisation of Biological Products and Vaccines, Helwan, Cairo, Egypt. The animals were kept in plastic cages at 25 °C with 12 h light/dark cycles [51]. For 28 days, animals were provided unrestricted access to water and a baseline feed formulated according to the AIN-93 recommendations [52].

#### 2.8.2 Ethical aspects

The rules for the care and utilization of experimental animals were approved by the Ethics Committee of Zagazig University under Protocol No. ZUIACUC/3/F/175/2019.

#### 2.8.3 Design of an experiment

After 15 days of acclimation, the rats were divided into eight groups (G1–G8). Each group consisted of five rats.

**Table:** 

G1:	The control group consisted of rats that were given a vehicle and observed for 28 days.
**G2:**	Carbon tetrachloride (CCl_4_) was injected intraperitoneally into rats twice weekly for 28 days at a dose of 0.3 mL/kg BW [12].
**G3:**	The rats received MO leaf aqueous extract at a dosage of 100 mg/kg BW each day, along with oral administration of CCl_4_, for 28 days [12].
**G4:**	The rats received MO leaf aqueous extract at a dosage of 200 mg/kg BW each day, along with oral administration of CCl_4_, for 28 days [12].
**G5:**	The rats were given chemical ZnO-NPs at a dosage of 100 mg/kg BW every day, along with oral administration of CCl_4_, for 28 days [35].
**G6:**	The rats received chemical ZnO-NPs at a dosage of 200 mg/kg BW every day, along with oral administration of CCl_4_, for 28 days [35].
**G7:**	The rats were given green synthesized ZnO-NPs at a dosage of 100 mg/kg BW every day, along with oral administration of CCl_4_, for 28 days.
**G8:**	The rats received green synthesized ZnO-NPs at a dosage of 200 mg/kg BW every day, along with oral administration of CCl_4_, for 28 days.

#### 2.8.4 The gathering of blood and tissue samples

Following the experiment, blood samples were collected from animals that had undergone overnight fasting while being exposed to tetrahydrofuran inhalation anesthesia. Then, blood samples from the retroorbital venous plexus were drawn into sterile, dry centrifuge tubes to separate the serum at 2000×*g* for 15 min at 4 °C. Subsequently, the liver was extracted, put on filter paper, and washed with cold phosphate buffer saline. It was also partitioned into two distinct groups. In the context of histopathology, the initial batch of samples was immersed in a 10 % neutral buffer formalin solution. To determine the levels of antioxidant enzymes, the second group was kept at −80 °C [53].

#### 2.8.5 Measurement of liver function, renal function, and lipid profile

Following the manufacturer's instructions, the levels of various serum markers, including ALT, ALP, AST, LDH, GGT, total proteins (TP), albumin (Alb), urea, uric acid, creatinine, blood urea nitrogen (BUN), triglycerides (TG), total cholesterol (TC), and HDL-C, were determined using a commercially available kit obtained from Bio Diagnostic Co., 29 El-Tahrir St. Dokki, Giza, Egypt. Serum globulin was calculated using the following equation [54]:

Globulin=TP−Alb.

LDL-C levels were estimated using the following equation [55]:

LDL−C=TC−[HDL+VLDL].

VLDL was determined using the following equation:

VLDL=TG/5.

#### 2.8.6 Antioxidant markers in the liver

The liver was promptly rinsed with an ice-cold saline solution to eliminate any residual blood. Liver tissue was homogenized in 1:9 (w/v) cold potassium phosphate saline (0.1 M, pH = 7.4) before being centrifuged at 5000×*g* for 10 min at 4 °C. Then, the supernatant was used to estimate antioxidant enzymes [51]. The contents of malondialdehyde (MDA), GPx, GST, SOD, and GSH in the supernatant were quantified using colorimetric test kits obtained from Biodiagnostic Co. Subsequently, the experiments were completed following the manufacturer's instructions.

#### 2.8.7 Hematology

A full blood count was performed using the SYSMEX hematology auto analyzer (Japan), estimating white blood cells, platelets, red blood cells, hemoglobin concentration, hematocrit, mean corpuscular hemoglobin, mean corpuscular volume, mean corpuscular hemoglobin concentration, lymphocytes, neutrophils, eosinophils, and monocytes.

#### 2.8.8 The examination of the liver under a microscope

Slices of 4–5 mm thickness were cut from paraffin blocks containing the preserved liver tissues. To check for CCl_4_-related liver damage, we stained them with hematoxylin and eosin (H&E) and examined them under a microscope at magnifications between 100× and 400x.

### 2.9 Statistical analysis

The SPSS for Windows v. 11.0 (SPSS Ltd., Surrey, UK) was used to conduct the statistical analysis, and the obtained findings were presented as mean ± SD. A one-way analysis of variance (ANOVA) with Tukey's post hoc test was used to compare the groups across all parameters of interest. The statistical significance was set at p < 0.05.

## 3 Results

### 3.1 Phytochemical screening of MO

A phytochemical screen or qualitative analysis is conducted to determine the constituent chemicals and secondary metabolites in MO leaves. The initial phytochemical screening of MO leaves revealed the existence of phenolics, steroids, terpenoids, flavonoids, alkaloids, tannins, and saponins (Table 1). The MO leaves contained all secondary metabolites. The leaves were composed of 7.4 % phenolic compounds, 6.8 % flavonoids, 5.1 % steroids, 5.4 % terpenoids, 12.2 % tannins, and 6.2 % saponins.

**Table 1:** Phytochemical screening of *Moringa oleifera* leaves.

No.	Phytoconstituents	Quantity (%)
1	Phenolic compound	7.4
2	Flavonoids	6.8
3	Steroids	5.1
4	Terpenoids	5.4
5	Alkaloids	4.2
6	Tannins	12.2
7	Saponins	6.2

### 3.2 Moringa oleifera leaf aqueous extract characterization

#### 3.2.1 TPC and TFC

An aqueous extract of MO leaves was produced, and the Folin–Ciocalteu reagent test was employed to ascertain the TPCs. The aluminum chloride method was used to quantify the TFs present in the identical sample. Table 2 displays the extract yield, TPCs (GAE/g DE), and TFs (QE/g DE) for the MO aqueous extract. The yield of extraction was 15 g/100 g of dry leaves. MO aqueous extract had a TPC content of 171.67 mg GAE/g DE and a TF content of 82 mg QE/g DE. Based on earlier research, an aqueous extract of MO leaf was discovered to be a valuable source of active ingredients such as phenolics and flavonoid compounds.

**Table 2:** Yield of extract (g), TPCs (GAE g^−1^ dry extract), and TFs (QE g^−1^ dry extract).

Parameters	Concentration
Yield of extract	15 g/100g dry weight
TPCs	171.67 mg GAE g^−1^ dry extract
TFs	82 mg QE g^−1^ dry extract

TPCs: total phenolic compounds; TFs: total flavonoids.

#### 3.2.2 Quantification of phenolic compounds by HPLC

**Fig. 2:**
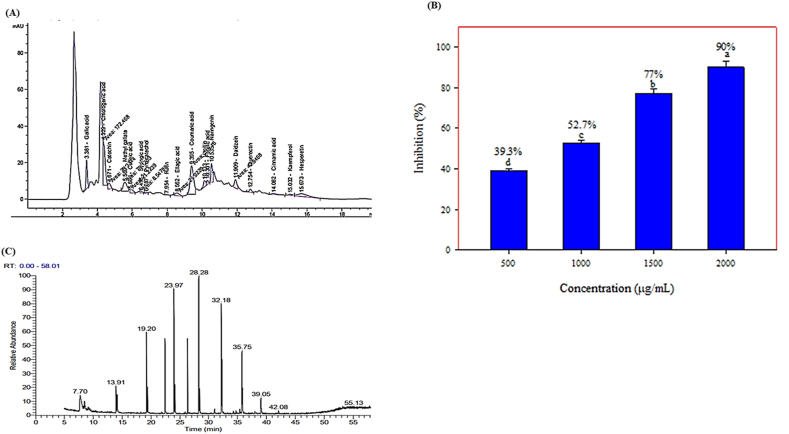
**(A)** HPLC-chromatogram of major phenolic compounds in MO aqueous extract. The phenolic compounds were detected via comparison to an external standard retention time; **(B)** Antioxidant activity (% inhibition) of MO leaf extract at different concentrations (500, 1000, 1500, and 2000 μg/mL), and **(C)** GC-MS chromatogram of moringa leaf aqueous extract.

Measuring the total phenolic content through spectrophotometric methods does not provide a complete understanding of the quality or quantity of phenolic components present in the extract. Therefore, phenolics present in the aqueous extract of MO leaves were identified using HPLC analysis. Fig. 2A depicts a chromatogram that exemplifies the examination of phenolic constituents in the aqueous extract of MO using HPLC. In Fig. 2A of the chromatogram, the aqueous extract of MO exhibited a total of 18 prominent peaks. The MO aqueous extract contains phenolic compounds 1–19 (Table 3). Moreover, the extract contains chlorogenic, ellagic, and coumaric acids in the highest concentrations of 1384.33 μg/g, 612.58 μg/g, and 522.72 μg/g, respectively. Previous research revealed the presence of phenolic acids, including gallic acid (381.54 μg/g), catechin (449.56 μg/g), chlorogenic acid (1384.33 μg/g), and ellagic acid (612.58 μg/g), and flavonoids, such as quercetin (137.81 μg/g), quercitrin (131.89 μg/g), kaempferol (83.10 μg/g), naringenin (407.38 μg/g), hesperetin (200.67 μg/g), daidzein (203.87 μg/g), and rutin (8.46 μg/g) in MO leaf extract.

**Table 3:** Major phenolic compounds (RT) retention time (min), area (%), and concentration (μg/g extract) in *Moringa oleifera* aqueous extract.

No.	RT	Phenolic compound	Conc. (μg/g)
1	3.381	Gallic acid	381.54
2	4.329	Chlorogenic acid	1384.33
3	4.671	Catechin	449.56
4	5.58	Methyl gallate	293.55
5	5.868	Coffeic acid	96.04
6	6.496	Syringic acid	41.57
7	6.787	Pyro catechol	62.64
8	7.954	Rutin	8.46
9	8.562	Ellagic acid	612.58
10	9.395	Coumaric acid	522.72
11	10.13	Vanillin	100.85
12	10.301	Ferulic acid	62.81
13	10.535	Naringenin	407.38
14	11.909	Daidzein	203.87
15	12.754	Quercetin	131.89
16	14.082	Cinnamic acid	12.70
17	14.515	Apigenin	0.00
18	15.032	Kaempferol	83.10
19	15.673	Hesperetin	200.67

#### 3.2.3 Antioxidant activity (DPPH-assay)

Measuring the antioxidant activity of MO aqueous leaves using the 2,2-diphenyl-1-picrylhydrazyl (DPPH) assay is necessary to complete the data. The antioxidant activity of the MO leaf extract was assessed using the DPPH test at various concentrations, including 500, 1000, 1500, and 2000 μg/mL, and the results are presented in Fig. 2B. With an increase in the concentration of MO leaf extract, the antioxidant activity correspondingly increased. The efficacy of DPPH radical scavenging exhibited a notable increase from 39.3 % to 90 % as the concentration of the extract increased from 500 to 2000 μg/mL. The prior findings confirmed our beliefs that plant extracts' polyphenolic components are responsible for their antioxidant actions. These chemicals can be efficient antioxidants. Furthermore, it is well-known that plant extracts containing phenolic and flavonoid compounds have high antioxidant content. The SC_50_ value was determined as the concentration of the sample needed to lower DPPH concentration by 50 %. The SC_50_ value for the MO leaf aquatic extract was 940 μg/mL.

#### 3.2.4 GC-MS analysis

The aqueous fraction of MO leaf extract was performed using gas GC-MS analysis, which detected 28 distinct peaks and depicted the presence of 28 bioactive compounds. Fig. 2C illustrates a chromatogram, whereas Table 4 lists the bioactive compounds, together with their corresponding retention time (RT), peak areas expressed as percentages, molecular formula, and molecular weight (MW). Among the 28 compounds identified, the major compounds present in *M. oleifera* were 2-hexynoic acid; 1,3-dioxolan-2-1,4,5-dimethyl-; d-mannoheptulose; thymol derivative; tetraacetyl-*d*-xylonic nitrile; and 3-[1-(4-cyano-1,2,3,4 tetrahydronaphthyl)] propanenitrile; and other compounds were identified as low level.

**Table 4:** GC-MS analysis of *Moringa Oleifera* leaf extract.

PN	RT	Active compounds	MW	MF	Area %
1	7.70	1-Nitro-2-acetamido-1,2-dideoxy-*d*-glucitol	252	C_8_H_16_N_2_O_7_	2.61
2	8.97	1,3-Dihydroxyacetone dimer	180	C_6_H_12_O_6_	3.82
3	10.65	Acetic acid, [(aminocarbonyl)amino]oxo-	132	C_3_H_4_N_2_O_4_	3.21
4	11.49	Trehalose	342	C_12_H_22_O_11_	2.23
5	13.91	4(1H)-Pyrimidinone, 2,6-diamino-	126	C_4_H_6_N_4_O	2.23
6	16.73	4H-Pyran-4-one, 2,3-dihydro-3,5-dihydroxy-6-methyl-	144	C_6_H_8_O_4_	1.12
7	19.2	2-Hexynoic acid	112	C_6_H_8_O_2_	6.12
8	20.89	Butanedioic acid, 2-hydroxy-2-methyl-, (S)-	148	C_5_H_8_O_5_	3.14
9	21.38	3,3′-Methyliminobispropylamine	145	C_7_H_19_N_3_	1.92
10	21.68	1-Hexanamine	101	C_6_H_15_N	0.16
11	23.97	1,3-Dioxolan-2-one, 4,5-dimethyl-	116	C_5_H_8_O_3_	12.32
12	24.31	2-Butenethioic acid, 3-(ethylthio)-, S-(1-methylethyl) ester	204	C_9_H_16_OS_2_	1.31
13	25.57	Propanamide, N,N-dimethyl-	101	C_5_H_11_NO	3.12
14	26.33	Anthracene, 9-(2-propenyl)	218	C_17_H_14_	2.13
15	27.11	2-Isopropoxyethyl propionate	160	C_8_H_16_O_3_	1.53
16	28.28	d-Mannoheptulose	210	C_7_H_14_O_7_	18.13
17	28.76	Thymol, derivative	222	C_13_H_22_N_5_Si	4.63
18	31.32	Carbonic acid, butyl 2-pentyl ester	188	C_10_H_20_O_3_	2.56
19	32.18	Tetra acetyl-*d*-xylonic nitrile	343	C_14_H_17_NO_9_	8.84
20	33.15	alpha-d-Glucose	180	C_6_H_12_O_6_	3.44
21	34.11	1H-Cyclopenta[*c*]furan-3(3aH)-one,6,6a-dihydro-1-(1,3-dioxolan-2-yl)-,(3aR,1-trans,6a-cis)-	196	C_10_H_12_O_4_	1.26
22	35.75	3-[1-(4-Cyano-1,2,3,4-tetrahydronaphthyl)]propanenitrile	210	C_14_H_14_N_2_	5.12
23	39.05	Quinolinium, 1-ethyl-, iodide	285	C_11_H_12_IN	4.31
24	40.03	N-Isopropyl-3-phenylpropanamide	191	C_12_H_17_NO	1.12
25	40.22	Propanamide	73	C_3_H_7_NO	0.73
26	41.89	1,2-Ethanediamine, N-(2-aminoethyl)-	103	C_4_H_13_N_3_	0.88
27	42.91	1,4-Benzenediol, 2-methyl-	124	C_7_H_8_O_2_	1.42
28	43.05	Ethene, ethoxy-	72	C_4_H_8_O	0.61
Total area percent (%)					100.02

PN = peak number; RT = retention time (minit); MF = molecular formula; MW = molecular weight.

### 3.3 Green and chemically synthesized ZnO-NP characterization

#### 3.3.1 ZnO-NPs characterization

**Fig. 3:**
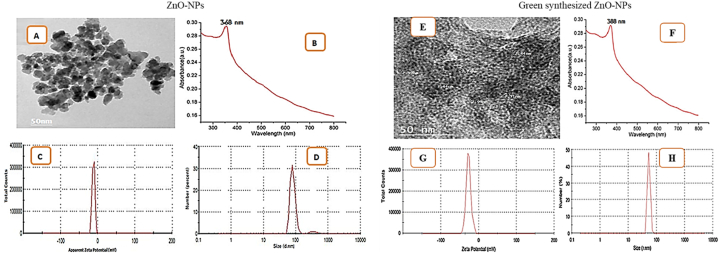
Transmission electron microscope **(A)**, absorbance of ZnO-NPs **(B)** zeta potential **(C)**, and particle size **(D)** ZnO-NPs prepared using chemical method and transmission electron microscope **(E)**, absorbance of ZnO-NPs **(F)** zeta potential **(G)**, and particle size **(H)** of ZnO-NPs green synthesis by*Moringa oleifera* aqueous leaf extract.

In the TEM image (Fig. 3A), chemical analysis of ZnO-NPs revealed a well-dispersed, almost spherical form with a particle size of around 85 nm. The room temperature UV–visible absorption spectra of chemically produced ZnO-NPs range from 300 to 800 nm (Fig. 3B). The spectrum shows a peak at 368 nm (3.38 eV). The moderately stable NPs' zeta potential of −11.5 mV was utilized to calculate stability and surface charge (Fig. 3C). Furthermore, an additional method of confirmation was employed utilizing DLS to evaluate NPs’ dispersion and ascertain their size. The results revealed a limited range of sizes, with an average particle size of 80 ± 5 nm as a first peak, with some aggregates (clusters) emerging in a very tiny percentage as a second peak ranging from 100 to 1000 nm with PDI = 0.552, indicating a narrow size range (Fig. 3D).

#### 3.3.2 Characterization of greenly synthesized ZnO-NPs prepared by MO leaf aqueous extract

Several characterization methodologies, including TEM, UV-optical absorption zeta potential, and zeta size, were used to determine the synthesis of green-produced ZnO-NPs by MO leaf aqueous extract. TEM was employed to investigate the size and form of biosynthesized ZnO-NPs (Fig. 3E). Greenly synthesized ZnO-NPs UV–Vis spectra revealed a notable peak at 388 nm (Fig. 3F). This expresses that ZnO-NPs exhibit excitation absorption (at 388 nm) due to their vast excitation binding energy at 37 °C, demonstrating that the MO leaf aqueous extract competently reduces the Zn ion. The zeta potential (Fig. 3G) and zeta size (Fig. 3H) analyses supported the NP's average size and stability. These results displayed that the zeta potential was −41.7 mv and the mean diameter of ZnO-NPs was around 55 nm.

### 3.4 Hepatoprotective impact

#### 3.4.1 Serum enzymes

**Fig. 4:**
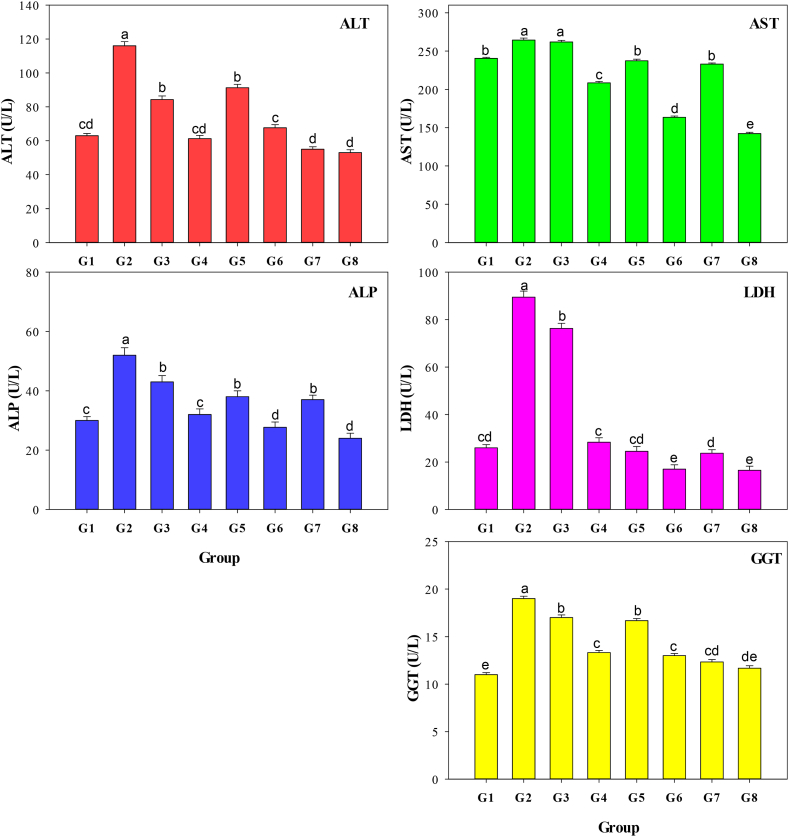
Serum alanine aminotransferase (ALT), aspartate aminotransferase (AST), alkaline phosphatase (ALP), lactate dehydrogenase (LDH), gamma-glutamyl transferase (GGT) of albino rats receiving the same level of CCl_4_ as the positive control but treated with two levels of *Moringa oleifera* aqueous extract 100 (G3) and 200 (G4) mg/kg BW every day, two levels of ZnO-NPs 100 (G5) and 200 (G6) mg/kg BW every day, and two levels of greenly synthesized ZnO-NPs 100 (G7) and 200 (G8) mg/kg BW every day compared with the negative (G1) and positive (G2) controls. Different letters indicate significant differences (*P* < 0.001). Error bars represent standard deviations.

Initially, an examination was conducted to assess the impact of MO leaf aqueous extract, greenly synthesized ZnO-NPs, and ZnO-NPs on the hepatic function of albino rats treated with CCl_4_. This was accomplished by evaluating the values of serum enzymes of ALT, ALP, AST, LDH, and GGT. The outcomes of this investigation are displayed in Fig. 4. Compared to the G1 group, the group of rats intoxicated with CCl_4_ (G2) exhibited a significant rise in activity levels of liver enzymes AST, ALP, ALT, LDH, and GGT (*P* < 0.001). Oral treatment of MO leaf aqueous extract, greenly synthesized ZnO-NPs, and ZnO-NPs at two doses effectively prevented the increase in the five biomarkers when compared to positive control (G2) (*P* < 0.001). Regarding AST activity, nonsignificant changes (*P* < 0.001) were observed in G3 rats who were given a dosage of 100 mg/kg BW of MO leaf aqueous extract compared to the rats who were intoxicated with CCl_4_. Groups G7 and G8 received greenly produced ZnO-NPs at two doses, which significantly influenced liver enzyme activity compared to other treatments (MO leaf aqueous extract and ZnO-NPs). This research indicates that MO leaf aqueous extract, ZnO-NPs, and greenly synthesized ZnO-NPs can normalize liver function and activity.

#### 3.4.2 Protein profile

**Fig. 5:**
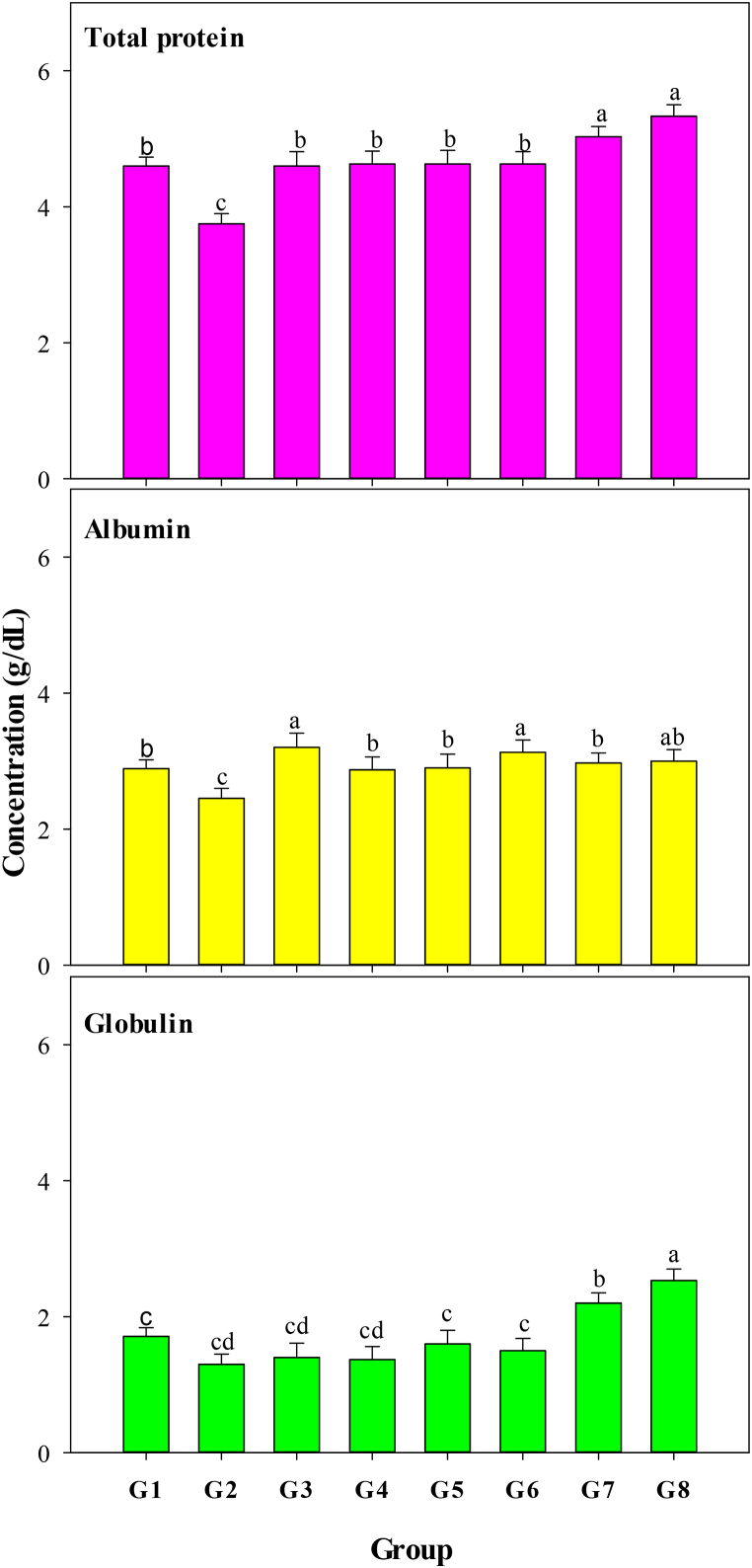
Total protein, albumin, and globulin of albino rats receiving the same level of CCl_4_ as the positive control but treated with two levels of *Moringa oleifera* aqueous extract 100 (G3) and 200 (G4) mg/kg BW every day, two levels of ZnO-NPs 100 (G5) and 200 (G6) mg/kg BW every day, and two levels of greenly synthesized ZnO-NPs 100 (G7) and 200 (G8) mg/kg BW every day compared with the negative (G1) and positive (G2) controls.

The impacts of MO leaf aqueous extract, ZnO-NPs, and greenly synthesized ZnO-NPs on the liver functions of CCl_4_-treated albino rats were evaluated by investigating TP, albumin, and globulin levels, and the results are illustrated in Fig. 5. TP (*P* < 0.008) and albumin (*P* < 0.015) levels in the positive control (G2) were substantially lower than those in the negative control group (G1). MO leaf aqueous extract was used in groups G3 (100 mg/kg BW every day) and G4 (200 mg/kg BW every day); ZnO-NPs in groups G5 (100 mg/kg BW every day) and G6 (200 mg/kg BW every day); and greenly synthesized ZnO-NPs in groups G7 (100 mg/kg BW every day) and G8 (200 mg/kg BW every day). Total protein levels were significantly (*P* < 0.008) higher in greenly synthesized ZnO-NPs at 100 (G7) and 200 (G8) mg/Kg BW in groups G3 (100 mg/kg BW every day) and G4 (200 mg/kg BW every day), as well as ZnO-NPs in groups G5 (100 mg/kg BW every day) and G6 (200 mg/kg BW every day). Groups G3 and G4, which received MO leaf aqueous extract at doses of 100 mg/kg BW every day and 200 mg/kg BW every day, respectively, and groups G5 and G6, which received ZnO-NPs at doses of 100 mg/kg BW every day and 200 mg/kg BW every day, showed no significant differences (*P* < 0.008) when compared to the negative control group (G1). Albumin levels were significantly higher in G3, G6, and G8 (*P* < 0.015) than those in G4, G5, and G7, as well as the negative control (G1). Moreover, globulin levels in groups G7 and G8, which received green synthesized ZnO-NPs at two doses, were significantly increased (*P* < 0.001) when compared to other treatments (G3, G4, G5, and G6), as well as negative (G1) and positive (G2) controls. According to the findings, the aqueous extract of MO leaves, as well as both ZnO-NPs and greenly synthesized ZnO-NPs, can restore normal liver function and activity in CCl_4_-treated albino rats by enhancing total protein and albumin levels.

#### 3.4.3 Lipid profile

**Fig. 6:**
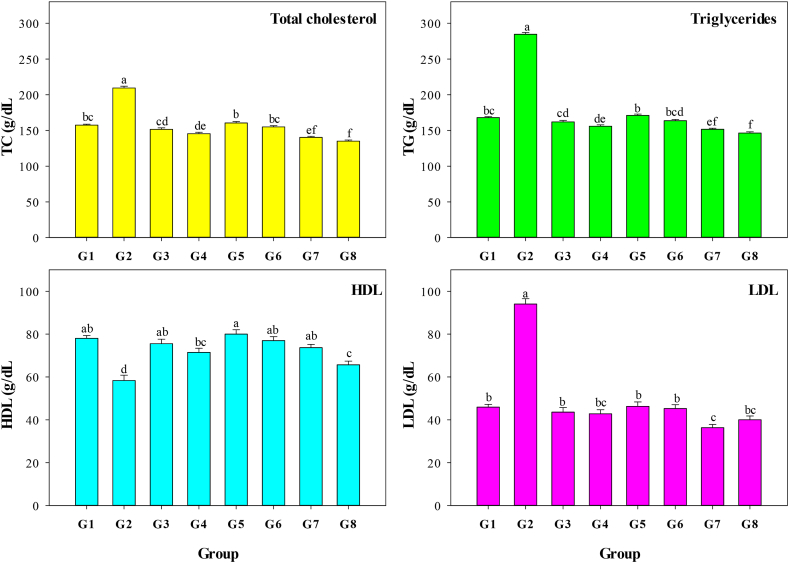
Total cholesterol (TC), triglycerides (TG), low-density lipoprotein (LDL), and high-density lipoprotein (HDL) of albino rats receiving the same level of CCl_4_ as the positive control but treated with two levels of *Moringa oleifera* aqueous extract 100 (G3) and 200 (G4) mg/kg BW every day, two levels of ZnO-NPs 100 (G5) and 200 (G6) mg/kg BW every day, and two levels of greenly synthesized ZnO-NPs 100 (G7) and 200 (G8) mg/kg BW every day compared with the negative (G1) and positive (G2) controls.

The effects of MO leaf aqueous extract, greenly synthesized ZnO-NPs, and ZnO-NPs on the lipid profile of CCl_4_-treated albino rats were investigated. TG, TC, HDL, and LDL levels were measured, and the findings are displayed in Fig. 6. The G2 group had significantly greater levels of TG, TC, and LDL (*P* < 0.001) than G1 (negative control group).

By administering MO leaf aqueous extract at two doses (G3 and G4), ZnO-NPs at two doses (G5 and G6), and greenly synthesized ZnO-NPs at two doses (G7 and G8), TC, TG, and LD levels were significantly reduced (*P* < 0.001) compared with those in the positive control group (G1). The highest impact on enhancing TG, TC, and LDL levels was recorded for groups G7 and G8 that received greenly synthesized ZnO-NPs (100 and 200 mg/kg BW, respectively) compared to other treatments (MO leaf aqueous extract and ZnO-NPs). In contrast, the G2 group had significantly (*P* < 0.001) lower HDL levels compared to the G1 group. Adverse effects of CCl_4_ on HDL levels were reduced by administering MO leaf aqueous extract at doses of 100 mg/kg and 200 mg/kg to groups G3 and G4, respectively, as well as ZnO-NPs in groups G5 (100 mg/kg BW every day) and G6 (200 mg/kg BW every day) and greenly synthesized ZnO-NPs in groups G7 (100 mg/kg BW every day) and G8 (200 mg/kg BW every day). Based on these results, it appears that using MO leaf aqueous extract, ZnO-NPs, and greenly synthesized ZnO-NPs can help protect albino rats from the negative effects of CCl_4_ by enhancing their lipid profile (including TC, TG, HDL, and LDL).

#### 3.4.4 Biomarkers for kidney function

**Fig. 7:**
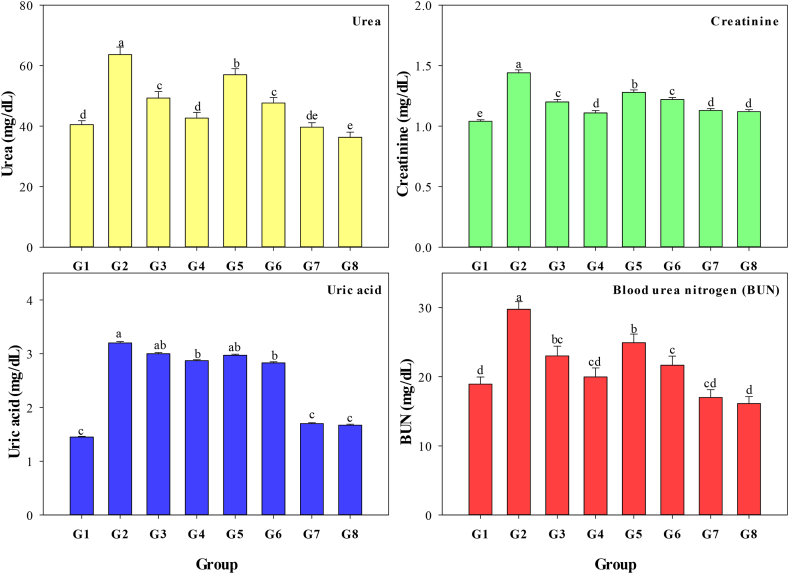
Serum urea, creatinine, uric acid, and blood urea nitrogen (BUN) of albino rats receiving the same level of CCl_4_ as the positive control but treated with two levels of *Moringa oleifera* aqueous extract 100 (G3) and 200 (G4) mg/kg BW every day, two levels of ZnO-NPs 100 (G5) and 200 (G6) mg/kg BW every day, and two levels of greenly synthesized ZnO-NPs 100 (G7) and 200 (G8) mg/kg BW every day compared with the negative (G1) and positive (G2) controls.

The current research examines the impact of an aqueous extract of MO leaves, ZnO-NPs, and greenly synthesized ZnO-NPs on renal function in albino rats treated with CCl_4_. Evaluation of kidney function is based on serum urea, uric acid, creatinine, and BUN measurements, and corresponding results are presented in Fig. 7. Compared to the negative control group (G1), the positive control group (G2) demonstrated a statistically significant elevation in serum concentrations of urea (*P* < 0.001), uric acid (*P* < 0.001), creatinine (*P* < 0.007), and BUN (*P* < 0.001). Providing MO leaf aqueous extract, greenly synthesized ZnO-NPs, and ZnO-NPs at two doses was discovered to significantly inhibit the rise in variables such as urea, uric acid, creatinine, and BUN. The green ZnO-NPs, given in two doses, efficiently maintained normal levels of renal variables such as urea, uric acid, and BUN. According to the findings, the aqueous extract of MO leaves, as well as ZnO-NPs (both greenly synthesized and regular), could potentially aid in restoring kidney function and activity.

#### 3.4.5 Biomarkers for hepatic oxidative stress

**Fig. 8:**
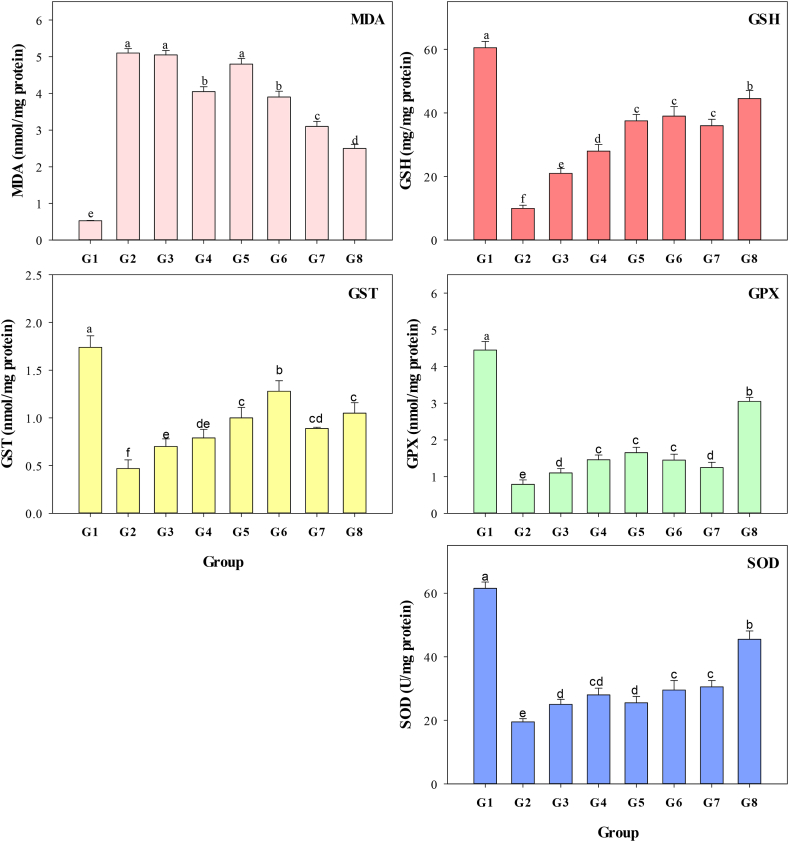
Malondialdehyde (MDA), glutathione peroxidase (GPx), glutathione-S-transferase (GST), superoxide dismutase (SOD), and glutathione (GSH) of albino rats receiving the same level of CCl_4_ as the positive control but treated with two levels of *Moringa oleifera* aqueous extract 100 (G3) and 200 (G4) mg/kg BW every day, two levels of ZnO-NPs 100 (G5) and 200 (G6) mg/kg BW every day, and two levels of greenly synthesized ZnO-NPs 100 (G7) and 200 (G8) mg/kg BW every day compared with the negative (G1) and positive (G2) controls.

Fig. 8 depicts the findings of testing hepatic oxidative stress indicators (MDA, GSH, GST, GPX, and SOD) to determine whether MO leaf aqueous extract, greenly produced ZnO-NPs, and ZnO-NPs could protect CCl_4_-treated albino rats. Based on these outcomes, CCl_4_ (G2) was observed to increase MDA significantly (P < 0.001) compared to negative control (G1). MO leaf aqueous extract at 200 mg/kg BW (G4), ZnO-NPs at 200 mg/kg BW (G6), and greenly synthesized ZnO-NPs at two doses (G7 and G8) significantly reduced lipid peroxidation (*P* < 0.001). However, employing MO leaf aqueous extract at a daily dosage of 100 mg/kg BW (G3) and ZnO-NPs at a daily dosage of 100 mg/kg BW (G5) had no significant effect on MDA levels (*P* < 0.001). Compared to G1, G2 exhibited considerably lower GPx, GST, SOD, and GSH levels (*P* < 0.001). The levels of vital antioxidant enzymes in CCl_4_-stressed albino rats were restored by administering two dosages of MO leaf aqueous extract (G3 and G4), ZnO-NPs (G5 and G6), and greenly synthesized ZnO-NPs (G7 and G8). Rats given a larger dose of greenly synthesized ZnO-NPs (G8) had significantly higher GSH, GPX, and SOD levels (*P* < 0.001). Rats received 200 mg/kg BW every day of ZnO-NPs and had the highest GST reported value. Based on our findings, the use of MO leaf aqueous extract, greenly synthesized ZnO-NPs, and ZnO-NPs has been shown to defend against hepatic injury in albino rats treated with CCl_4_. This is achieved by improving and increasing the levels of hepatic oxidative stress biomarkers, such as GST, GSH, CAT, GPX, and SOD, while decreasing MDA levels.

#### 3.4.6 Hematological parameters

Specific hematological parameters were evaluated to confirm the hepatoprotective impact of MO leaf aqueous extract, greenly synthesized ZnO-NPs, and ZnO-NPs, in CCl4-treated rats, and the findings are listed in Table 5. When comparing G1 to G2, it was observed that CCl_4_ had a substantial impact on reducing RBC, HCT, MCV, Hb, MCH, MCHC, and Plt levels. Compared to nontreated groups, the administration of an aqueous extract of MO leaf, ZnO-NPs, and greenly synthesized ZnO-NPs at dosages of 100 and 200 mg/kg BW per day resulted in a substantial prevention of a drop in RBC, HCT, MCV, Hb, MCH, MCHC, and Plt levels in treatment groups. In contrast, the group treated with CCl_4_ (G2) showed a notable rise in levels of WBC, neutrophils, and monocytes when compared to the negative control group (G1). By administering MO leaf aqueous extract at doses of 100 mg/kg BW every day and 200 mg/kg BW every day to groups G3 and G4, respectively, ZnO-NPs at 100 mg/kg BW every day and 200 mg/kg BW every day to groups G5 and G6, respectively, and greenly synthesized ZnO-NPs at 100 mg/kg BW every day and 200 mg/kg BW every day to groups G7 and G8, respectively, the levels of WBC, neutrophils, and monocytes were significantly declined compared to those in positive control (G1) and were restored to normal range. No statistically significant alterations in eosinophil levels were observed in any of the treatment groups when compared with positive and negative control groups.

**Table 5:** Hematological analysis including white blood cell (WBC), red blood cell (RBC), platelet counts (Plt), hemoglobin concentration (Hb), hematocrit (HCT), mean corpuscular volume (MCV), mean corpuscular hemoglobin (MCH), mean corpuscular hemoglobin concentration (MCHC), lymphocyte (LYM), neutrophils, eosinophils, and monocytes of albino rats receiving the same level of CCl_4_ as the positive control but treated with two levels of *Moringa oleifera* aqueous extract 100 (G3) and 200 (G4) mg/kg body weight/day, two levels of ZnO-NPs 100 (G5) and 200 (G6) mg/kg body weight/day, and two levels of greenly synthesized ZnO-NPs 100 (G7) and 200 (G8) mg/kg body weight/day compared with the negative (G1) and positive (G2) controls.

Parameters	Groups								*P-value*
	G1	G2	G3	G4	G5	G6	G7	G8	
WBC (10^3^/μL)	9.70 ^cd^	14.03^a^	12.47 ^b^	9.70 ^cd^	13.43 ^ab^	9.33 ^d^	10.80^c^	8.17 ^d^	0.019 *
RBC (10^6^/μL)	7.03 ^ab^	5.00^c^	7.00 ^ab^	6.63 ^b^	6.11 ^b^	7.05 ^ab^	6.61 ^b^	8.12^a^	0.036 *
Hb (g/dL)	14.30 ^bc^	13.23^c^	14.13^bc^	14.57 ^bc^	14.90 ^bc^	15.07 ^ab^	15.57 ^ab^	16.23^a^	0.016*
HCT (%)	45.55 ^b^	40.07 ^d^	43.77^c^	46.53 ^b^	45.50 ^b^	46.33 ^b^	49.10^a^	50.10^a^	0.014*
MCV (fL)	65.00^c^	57.33^e^	71.33 ^b^	73.33 ^b^	67.00^c^	76.33^a^	61.67 ^d^	77.33^a^	0.008 **
MCH (pg)	20.50 ^de^	19.00^e^	19.33^e^	22.00 ^bc^	21.67 ^cd^	23.67 ^b^	25.00^a^	25.67^a^	0.032 *
MCHC (g/dL)	33.00^a^	31.00 ^b^	31.50 ^b^	32.33 ^ab^	31.33 ^b^	32.67 ^ab^	33.00^a^	33.33^a^	0.021*
Plt (10^3^/μL)	922.33^a^	587.00^e^	706.50^c^	807.67 ^b^	632.33 ^d^	898.33^a^	795.00 ^b^	905.00^a^	0.005 **
Neutrophils (%)	23.93 ^bc^	30.57^a^	24.37 ^b^	25.07 ^b^	22.10 ^bc^	19.70^c^	17.17^c^	9.10 ^d^	0.009 **
Lymphocyte (%)	82.90^a^	64.17 ^b^	63.00 ^b^	67.57 ^b^	65.20 ^b^	64.47 ^b^	63.57 ^b^	55.93^c^	0.015 *
Monocyte (%)	6.80^e^	15.87^a^	10.14^cd^	8.78 ^d^	9.24 ^d^	13.46 ^b^	10.26 ^cd^	11.48^c^	0.008 **
Eosinophil (%)	0.800	1.867	1.193	1.033	1.087	1.583	1.207	1.350	0.181 NS

Different letters indicate significant differences.

#### 3.4.7 Liver histopathology

**Fig. 9:**
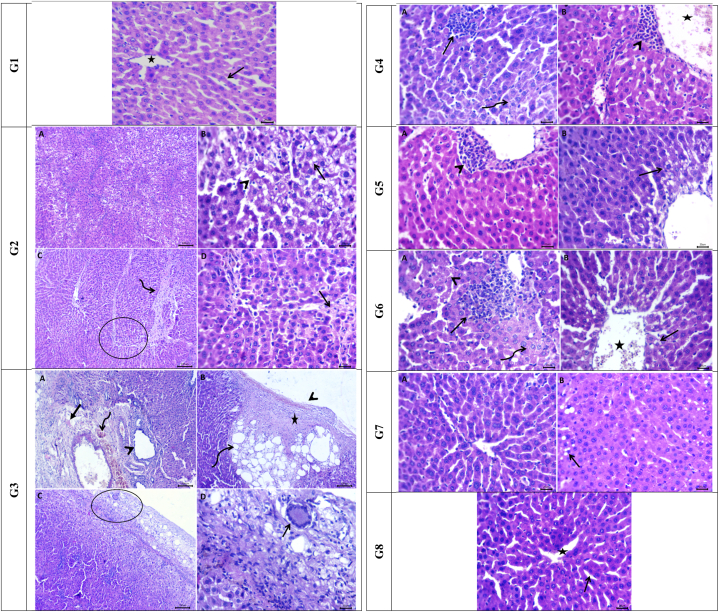
Photomicrographs of H&E-stained sections from the liver of rats in the negative control group (G1) show normal histological structures of the hepatic cords (arrow) and central vein (star). Scale bar: 20 μm, photomicrographs of H&E-stained sections from the liver of rats in the positive control group (G2). Numerous necrotic hepatocytes (arrowhead) are present alongside large areas of fatty changes (arrow) in the images of **A** and **B**. Scale bars are 100 and 20 μm, respectively. **C** and **D** show the presence of fibrotic bands between some hepatic lobules (curved arrow) with coagulative necrosis of some hepatocytes (arrow). Scale bars are 100 and 20 μm, respectively, photomicrographs of H&E-stained sections from the liver of rats in the positive control group (G2). In **A**, enlargement of the portal area by chronic inflammatory reactions with edema (closed arrow) and hemorrhages (curved arrow) beside the presence of hyperplastic epithelial lining the bile duct with a dilated lumen (arrowhead), Scale bar: 100 μm. **B** shows the thickening of hepatic capsules by chronic inflammatory reactions (star) admixed with superficial hemorrhages (star) alongside metaplastic changes of fibrous tissue into large fat cells (curved arrow). Scale bar: 100 μm. An arrow points to Touton giant cells in both **C** and **D**, which are visible within the thickened hepatic capsule. The scale bar for **C** is 100 μm, and for **D**, it is 20 μm. Photomicrographs of H&E-stained sections from the liver of albino rats receiving CCl_4_ and treated with 100 mg/kg BW every day of MO leaf aqueous extract (G3). In **A**, moderate amounts of hepatic parenchyma (curved arrow) and focal areas of interstitial round cell infiltrations (arrow) exhibit degenerative alterations. Scale bar: 20 μm. Image **B** displays crowded hepatic blood vessels (star) and accompanying perivascular round cell infiltrations (arrowhead). Scale bar: 20 μm, and photomicrographs of H&E-stained sections from the liver of albino rats receiving CCl_4_ and treated with 200 mg/kg BW every day of MO leaf aqueous extract (G4). In **A**, it shows perivascular lymphocytic aggregates (arrowhead). Scale bar: 20 μm. In **B**, fatty changes within some hepatocytes are shown (arrow). Scale bar: 20 μm, photomicrographs of H&E-stained sections from the liver of albino rats receiving CCl_4_ and treated with 100 **(A)** and 200 **(B)** mg/kg BW every day of ZnO-NPs (G5 and G6, respectively). In **A**, focal areas of inflammatory cell aggregates (arrow) are adjacent to hydropic degenerated cells (curved arrow) and minute fat droplets within some hepatic cells (arrowhead). Scale bar: 20 μm. **B** shows congested hepatic blood vessels (star) and fatty changes in a mild number of hepatocytes (arrow). Scale bar: 20 μm, photomicrographs of H&E-stained sections from the liver of albino rats receiving CCl_4_ and treated with 100 mg/kg BW every day of green synthesized ZnO-NPs (G7). In **A**, the hepatic structures appear normal (arrowhead). Scale bar: 20 μm. In **B**, fat globules are within a few numbers of hepatocytes (arrow). Scale bar: 20 μm, and photomicrographs of H&E-stained sections from the liver of albino rats receiving CCl_4_ and treated with 200 mg/kg BW every day of green synthesized ZnO-NPs (G8) showing the apparent normal cytoarchitecture of most hepatic parenchyma (arrow). Scale bar: 20 μm.

As part of the study, the liver was examined to corroborate the findings and detect any pathological changes. The liver histological structures of hepatic cords, central veins, and portal areas in the negative control group (G1) were found to be normal (Fig. 9 G1). In the positive control group (G2), the liver displayed significant fatty changes and numerous necrotic hepatocytes (Fig. 9 G2-A and G2-B). Upon examination, certain sections showed the presence of fibrotic bands between hepatic lobules, along with coagulative necrosis of some hepatocytes (Fig. 9 G2-C and G2-D). After analyzing certain sections of the liver, an enlargement of the portal areas was discovered due to chronic inflammatory reactions, along with the presence of edema and hemorrhages. Moreover, a hyperplastic epithelial lining bile duct with a dilated lumen was observed (Fig. 9 G3-A). Furthermore, chronic inflammation causing the thickening of the hepatic capsules was detected. Superficial hemorrhages were also present, along with fibrous tissue undergoing metaplastic changes into large fat cells. These cells extended toward the hepatic parenchyma and caused pressure atrophy (Fig. 9 G3-B). Some thickened capsules contained multinucleated giant cells known as “Touton giant cells” (Fig. 9 G3-C and G3-D). The livers of albino rats given CCl_4_ in combination with 100 mg/kg BW every day of MO leaf aqueous extract (G3) exhibited degenerative changes in the moderate number of hepatic parenchyma and isolated areas of interstitial round cell infiltrations (Fig. 9 G4-A). Additionally, hepatic blood vessel congestion and perivascular round cell infiltrations were observed (Fig. 9 G4-B). When albino rats received CCl_4_ and 200 mg/kg BW every day of MO leaf aqueous extract (G4), the liver developed perivascular lymphocytic aggregates (Fig. 9 G5-A) and fatty changes within some hepatocytes (Fig. 9 G5-B).

Livers of albino rats treated with CCl_4_ and 100 mg/kg BW every day of ZnO-NPs (G6) exhibited focal areas of inflammatory cell aggregates contiguous to hydropic degenerated cells and minute fat droplets within some hepatocytes (Fig. 9 G6-A). In contrast, liver sections from albino rats receiving CCl_4_ and 200 mg/kg BW every day of ZnO-NPs (G6) displayed congested hepatic blood vessels and fatty alterations in a small number of hepatocytes (Fig. 9 G6-B). Most of the hepatic parenchyma in liver sections from albino rats receiving CCl_4_ and treated with 100 mg/kg BW every day of green synthesized ZnO-NPs (G7) displayed seemingly normal hepatic structures (Fig. 9 G7-A), with the presence of fat globules within a small number of hepatocytes (Fig. 9 G7-B). The liver sections of albino rats administered CCl_4_ and 200 mg/kg BW every day of greenly synthesized ZnO-NPs (G8) exhibited normal cytoarchitectures in the majority of the hepatic parenchyma (Fig. 9 G8 and Table 6).

**Table 6:** Summary of the main histopathological lesion scores among different groups. The represented scores in the table are the mean lesion scores. Histologic lesions were scored for severity (-: absent; +: mild; ++: moderate; +++: severe).

Organ	Main lesions	G1	G2	G3	G4	G5	G6	G7	G8
Liver	Hydropic degenerated cells	–	++	++	+	++	+	+	+
	Fatty changes	–	+++	+	++	+	+	+	–
	Necrotic hepatocytes	–	+	–	–	–	–	–	–
	portal areas inflammation	–	+++	+	+	++	+	–	–
	Perihepatitis	–	+++	++	+	+	+	+	+
	Inflammatory cells aggregates	–	++	++	+	++	+	+	–
	Congested hepatic blood vessels		+	++	++	+	++	–	–

**G1:** Vehicle-treated rats kept on a normal diet and served as a control for 28 days; **G2:** Rats intoxicated with carbon tetrachloride (CCl_4_) at the dose level of 0.3 mL/kg body weight/twice a week intraperitoneally for 28 days; **G3:** Rats orally given *M. oleifera* extract at 100 mg/kg body weight/day and CCl_4_ as Group II for 28 days simultaneously; **G4:** Rats orally given *M. oleifera* extract at 200 mg/kg body weight/day and CCl_4_ as Group II for 28 days simultaneously; **G5:** Rats orally given ZnO-NPs at 100 mg/kg body weight/day and CCl_4_ as Group II for 28 days simultaneously; **G6**: Rats orally given ZnO-NPs at 200 mg/kg body weight/day and CCl_4_ as Group II for 28 days simultaneously; **G7:** Rats orally given greenly synthesized ZnO-NPs at 100 mg/kg body weight/day and CCl_4_ as Group II for 28 days simultaneously; **G8:** Rats orally given greenly synthesized ZnO-NPs at 200 mg/kg body weight/day and CCl_4_ as Group II for 28 days simultaneously.

## 4 Discussion

The analysis of phytochemicals in MO revealed the presence of alkaloids, flavonoids, saponins, sterols, and tannins within the extracted leaves [56]. Plant secondary metabolites, commonly referred to as phytoconstituents, represent a valuable and distinctive reservoir of food supplements and pharmaceuticals. Extensive studies documented their many functions and applications [57]. Alkaloids, sterols, saponins, and tannins have a considerable hepatoprotective impact [58]. Phytosterols and flavonoids have immunomodulatory, antioxidant, anticancer, and anti-inflammatory activities [56]. Furthermore, the primary phenolics and flavonoids detected by HPLC in the extract, such as catechin, kaempferol, and rutin, have antioxidant activity and hepatoprotective impacts [59,60].

**Fig. 10:**
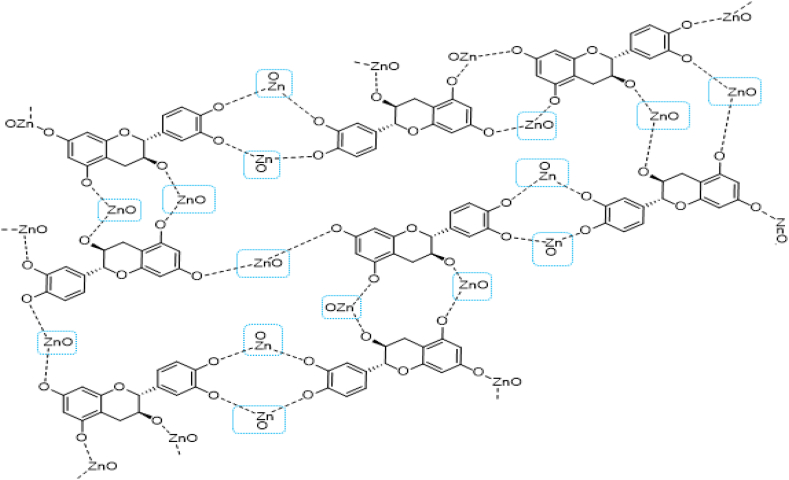
The schematic diagram illustrates the mechanism of zinc oxide nanoparticles (ZnO-NPs) formation from MO aqueous leaf extract.

Several methods can synthesize NPs; however, a green approach is increasingly preferred over traditional chemical and physical techniques. Synthesizing NPs using chemical and physical methods can be costly, time-consuming, and energy-intensive, negatively impact the environment, and result in hazardous substances on the surface. These NPs cannot be used for medical purposes [31,32]. In this study, ZnO-NPs were synthesized using the biological method. MO leaves aqueous extract was used as a reducing and stabilizing agent to synthesize ZnO-NPs as plant-based synthesis methods have many benefits, such as being easy to use, inexpensive, and achievable without utilizing natural solvents or harmful substances [33]. The phytochemical study of moringa extract confirms the presence of various unique compounds. In particular, this plant category is rich in phenolic compounds, which are used as reducing and agents to synthesize ZnO-NPs [34]. Whereas the precise method for producing ZnO-NPs from plant extracts is unknown, polar groups are thought to have a role [61]. Fig. 10 depicts one of the potential pathways for the plant extract's reducing and capping actions during ZnO-NP synthesis. According to the reduction of zinc nitrate utilizing MO extract as a biosynthesis of ZnO-NPs, compounds with (-OH, –NH, and NH2) can reduce the (Zn^2+^) ion. They have appropriate decreasing effects as well as strong stabilizing qualities during ZnONP preparation [[61], [62], [63]]. The chemical and green synthesis of ZnO-NPs has been extensively explored and confirmed using TEM, UV–Vis spectrophotometer, and DLS analysis. In the current study, the zeta potential was −41.7 mV and the mean diameter of ZnO-NPs was around 55 nm. These findings are similar to those of research that used moringa aqueous extract to create zinc oxide nanoparticles, albeit in different sizes [19,30,64].

The primary reason for doing this was to determine whether MO leaf aqueous extract, greenly synthesized ZnO-NPs, and ZnO-NPs could protect the liver of albino rats administered CCl_4_. Biochemical and histopathological assays are required to ascertain the toxicity of the numerous chemicals that serve as primary indicators of organ damage. Inducing liver damage with CCl_4_ and employing ALT, AST, ALP, LDH, and GGT as marker enzymes is a well-established science [65]. The hepatic enzyme ALT is essential for the metabolic conversion of proteins into energy within hepatocytes. In cases of liver injury, the ALT is produced and subsequently released into the circulatory system [66]. AST is an enzyme that helps in the metabolism of amino acids. AST, like ALT, is normally found in low levels in the blood. Elevated AST values may indicate liver damage, sickness, or muscular damage [67]. Moreover, ALP is an enzyme present in the liver and bone that aids in the breakdown of proteins. ALP levels higher than usual may suggest liver injury or disease, such as a blocked bile duct or certain bone diseases [68]. GGT is an enzyme present in blood. Abnormally high levels may indicate liver or bile duct injury [69]. Furthermore, the LDH test is primarily employed to determine the location and severity of tissue injury within the body [70]. First, the impacts of MO leaf aqueous extract, greenly synthesized ZnO-NPs, and ZnO-NPs on the liver function of CCl_4_-treated albino rats were investigated by assessing serum enzymes. The current study's reported elevations in blood AST, ALT, ALP, and LDH indicators in CCl_4_-treated rats may indicate hepatic cell injury. Furthermore, the findings on the impact of CCl_4_ intoxication on serum enzyme concentrations are consistent with those previously reported [51,71,72]. To safeguard the liver from the detrimental consequences of a hepatotoxin, any pharmaceutical agent designed for this purpose must be capable of either alleviating the adverse effects or reinstating the liver's customary physiological functioning after the ingestion of said hepatotoxin [73]. In this study, oral administration of MO leaf aqueous extract, greenly synthesized ZnO-NPs, and ZnO-NPs significantly suppressed increases in the five biomarkers. The lower amounts of ALT, AST, ALP, LDH, and GGT seen after ingestion of MO leaf aqueous extract may be attributable in part to the presence of bioactive components such as phenolics and flavonoids in the extract. At two levels (100 and 200 mg/kg BW every day), groups of greenly synthesized ZnO-NPs had the greatest impact on increasing the activity of liver enzymes. Measuring total protein levels and albumin-to-globulin ratios can aid in detecting various health issues, including liver disease [74]. Albumin is the most common protein in the blood, and it stops fluid from leaking and moving chemicals through the body [75]. Almost all other proteins in the blood are globulins, which are produced by the immune system and the liver [76]. Serum total protein and albumin concentrations decreased after CCl_4_ intoxication, and this may be possibly due to significant liver damage caused by lipid peroxidation, inflammation and/or breakdown of cellular membranes, and inactivation of synthetic activities. Trichloromethyl free radical-cell membrane conjugation may be to blame for the latter [77]. Liver damage caused by CCl_4_ can be attributed to a drop in total protein and albumin levels via increasing oxidative stress [78]. The TP levels in rats exposed to CCl_4_ were found to be reduced, suggesting the occurrence of hepatocyte lysis. This lysis resulted in a diminished capacity of the liver to perform protein synthesis, accompanied by an elevation in bilirubin concentration [79]. This study revealed that aqueous extract of MO leaf, ZnO-NPs, and greenly synthesized ZnO-NPs exhibited the ability to alleviate the adverse impacts of CCl_4_ on blood TP, albumin, and globulin levels by maintaining them within the normal physiological range. High amounts of TC, TG, and LDL were seen in the present study in CCI_4_-intoxicated rats. These findings are consistent with those in Abdul-Lattif [80] and Abu et al., [81]. This could be owing to the occurrence of liver damage. Possible causes include malfunctions in the systems that pair triglycerides with the proper apoprotein to make the transport molecule (lipoprotein) [82]. Albino rats exposed to CCl_4_ had their TC, TG, LDL, and HDL levels restored to normal range using treatment with MO leaf aqueous extract, greenly synthesized ZnO-NPs, and ZnO-NPs, suggesting that these NPs may facilitate oxidative reactions to preserve blood hemostasis. The treatment of CCl_4_-induced kidney toxicity is demonstrated by the rise in blood creatinine and urea [83]. Damage to the nephron's structure may potentially contribute to these pathological alterations [84], which is in line with research indicating that serum creatinine levels rise only in the presence of kidney impairment (specifically, damage to at least half of the kidney's nephrons) [85]. In the present study, CCl_4_-treated albino rats showed a considerable increase in serum amounts of urea, creatinine, uric acid, and BUN because of CCl_4_. The protective action of MO aqueous extract, ZnO-NPs, and greenly synthesized ZnO-NPs is not confined to the liver, but it is also effective in restoring or preserving renal physiology, as evidenced by the influence on serum urea, uric acid, creatinine, and BUN levels, particularly at elevated dosages. According to these findings, MO leaf aqueous extract, greenly synthesized ZnO-NPs, and ZnO-NPs all protect against oxidative stress and may protect various organ tissues. There are several ways in which biological systems guard themselves against the potentially disastrous impacts of activated species. SOD, GST, GSH, and GPX are cooperative defense mechanisms that prevent free radical injury to the body [[86], [87], [88], [89]]. In the positive control group, hepatic oxidative stress indicators (SOD, GST, GSH, and GPX) were reduced by CCl_4_-induced hepatotoxicity. This overall drop in antioxidant enzyme levels increased the oxidative stress on the liver, leading to hepatotoxicity [90]. Current research examines the impact of MO leaf aqueous extract, greenly synthesized ZnO-NPs, and ZnO-NPs on mitigating the significant decrease in antioxidant liver enzyme levels (SOD, GST, and GPX) and GSH caused by exposure to CCl_4_. This observed effect can be attributed to the overall protective mechanism of these agents in preserving the anabolic process, including enzyme biosynthesis. The present investigation observed elevated MDA levels in liver tissue homogenate of positive control group subjected to CCl_4_-induced hepatotoxicity. MDA, a significant consequence of lipid peroxidation, is a marker of oxidative damage and cellular injury [91,92]. Administration of these compounds by oral route reduced the elevation of MDA levels in rats that were injected with CCl_4_. This was achieved through inhibiting the lipid oxidation pathway, which is likely due to the possible antioxidant action of MO leaf aqueous extract, greenly synthesized ZnO-NPs, and ZnO-NPs. In this investigation, CCl_4_ considerably reduced RBC, Hb, HCT, MCV, MCH, MCHC, and Plt levels. In contrast, the levels of WBC, neutrophils, and monocytes were noticeably greater in the CCl_4_-treated group compared to those in the control group. The findings presented herein are consistent with those in prior research [[92], [93], [94], [95]]. By administering MO leaf aqueous extract, greenly synthesized ZnO-NPs, and ZnO-NPs, the levels of hematological parameters were adjusted in the normal range. The findings of the histology examinations supported the biochemical examination. The liver was studied to validate the findings and detect any abnormalities. Higher oxidative stress caused by CCl_4_ poisoning was most likely responsible for the hepatic tissue modifications observed in this study, including deformed hepatic plates, inflated hepatocytes with modest fatty changes, and thick portal tract mononuclear infiltration. Albino rats received 200 mg/kg BW every day of greenly synthesized ZnO-NPs immediately after CCl_4_ exposure and exhibited no major structural changes in the liver contrasted to positive control and other treatments, confirming the hepatoprotective ability of green synthesized ZnO-NPs. The presence of bioactive components including phenolics, tannins, saponins, flavonoids, alkaloids, and steroids in MO leaf aqueous extract may contribute to its hepatoprotective action. According to our obtained results, the highest impact on enhancing the hepatoprotective effect was recorded for groups G7 and G8 that received greenly synthesized ZnO-NPs. Furthermore, the administration of ZnO-NPs synthesized using green methods in groups G7 and G8 contributed to a greater level of protection than the usage of the aqueous extract of MO leaves. This can be attributed to the improved mechanism of drug transport.

## 5 Conclusions

In summary, the results derived from this investigation provide evidence supporting the protective properties of aqueous extract of MO leaves, ZnO-NPs, and greenly synthesized ZnO-NPs against liver damage induced by short-term exposure to CCl_4_ in rats. The direct antioxidant action against the free radicals (CCl3^•^ and CCl3OO^•^) produced is likely responsible for this protective effect. The highest impact on enhancing the hepatoprotective effect was recorded for rats that received greenly synthesized ZnO-NPs. Based on the findings, greenly synthesized ZnO-NPs in treatment provided a greater level of protection than MO leaf aqueous extract. This can be attributed to the improved method of drug administration.

## Funding

The research conducted in this study received support and funding from the 10.13039/501100011665Deanship of Scientific Research, Vice Presidency for Graduate Studies and Scientific Research, King Faisal University, Saudi Arabia (Grant 3972).

## Data availability statement

The datasets used and analyzed during the current study are available from the corresponding author upon reasonable request.

## CRediT authorship contribution statement

**Hossam S. El-Beltagi:** Writing – review & editing, Visualization, Validation, Supervision, Project administration, Funding acquisition, Formal analysis, Data curation, Conceptualization. **Marwa Rageb:** Writing – review & editing, Visualization, Software, Methodology, Formal analysis. **Mahmoud M. El-Saber:** Writing – original draft, Validation, Software, Resources, Methodology, Data curation, Conceptualization. **Ragab A. El-Masry:** Visualization, Validation, Supervision, Resources. **Khaled M.A. Ramadan:** Writing – review & editing, Visualization, Validation, Resources, Funding acquisition, Formal analysis. **Mahmoud Kandeel:** Writing – review & editing, Visualization, Validation, Resources, Funding acquisition. **Ahlam Saleh Alhajri:** Writing – review & editing, Visualization, Validation, Software, Resources, Funding acquisition. **Ali Osman:** Writing – review & editing, Writing – original draft, Visualization, Validation, Software, Project administration, Investigation, Formal analysis, Data curation, Conceptualization.

## Declaration of competing interest

The authors declare the following financial interests/personal relationships which may be considered as potential competing interests: Hossam El-Beltagi reports article publishing charges was provided by King Faisal University. If there are other authors, they declare that they have no known competing financial interests or personal relationships that could have appeared to influence the work reported in this paper.
